# Is deep brain stimulation a treatment option for anorexia nervosa?

**DOI:** 10.1186/1471-244X-13-277

**Published:** 2013-10-31

**Authors:** Marloes S Oudijn, Jitschak G Storosum, Elise Nelis, Damiaan Denys

**Affiliations:** 1 Department of Psychiatry, Academic Medical Centre, University of Amsterdam, Meibergdreef 5, 1105, AZ Amsterdam, the Netherlands; 2 Netherlands Institute for Neuroscience, an institute of the Royal Netherlands Academy of Arts and Sciences, Meibergdreef 47, 1105, BA Amsterdam, the Netherlands

**Keywords:** Anorexia nervosa, Deep brain stimulation, Compulsivity, Reward, Neurobiology

## Abstract

Anorexia nervosa (AN) is a severe psychiatric disorder with high rates of morbidity, comorbidity and mortality, which in a subset of patients (21%) takes on a chronic course. Since an evidence based treatment for AN is scarce, it is crucial to investigate new treatment options, preferably focused on influencing the underlying neurobiological mechanisms of AN. The objective of the present paper was to review the evidence for possible neurobiological correlates of AN, and to hypothesize about potential targets for Deep brain stimulation (DBS) as a treatment for chronic, therapy-refractory AN. One avenue for exploring new treatment options based on the neurobiological correlates of AN, is the search for symptomatologic and neurobiologic parallels between AN and other compulsivity- or reward-related disorders. As in other compulsive disorders, the fronto-striatal circuitry, in particular the insula, the ventral striatum (VS) and the prefrontal, orbitofrontal, temporal, parietal and anterior cingulate cortices, are likely to be implicated in the neuropathogenesis of AN. In this paper we will review the few available cases in which DBS has been performed in patients with AN (either as primary diagnosis or as comorbid condition). Given the overlap in symptomatology and neurocircuitry between reward-related disorders such as obsessive compulsive disorder (OCD) and AN, and the established efficacy of accumbal DBS in OCD, we hypothesize that DBS of the nucleus accumbens (NAc) and other areas associated with reward, e.g. the anterior cingulated cortex (ACC), might be an effective treatment for patients with chronic, treatment refractory AN, providing not only weight restoration, but also significant and sustained improvement in AN core symptoms and associated comorbidities and complications. Possible targets for DBS in AN are the ACC, the ventral anterior limb of the capsula interna (vALIC) and the VS. We suggest conducting larger efficacy studies that also explore the functional effects of DBS in AN.

## Introduction

Anorexia Nervosa (AN) is a severe psychiatric disorder characterized by an intense fear of gaining weight combined with a failure to maintain a minimally normal body weight (85% of the expected standard for age and height/ideal body weight). Patients with AN have a disturbed body image, are obsessed with weight and body shape, and are in a state of a denial with regard to their low body weight and its adverse impact on health (American Psychiatric Association 2006, 2000; Diagnostic and Statistical Manual of Mental Disorders 1994). In a subgroup of patients AN is associated with characteristic compulsive behaviors such as dieting, exercise, and/or purging with or without binge eating. Amenorrhea is often present in female patients but no longer required for the diagnosis (DSM 5, American Psychiatric Association 2013). With a narrow age of onset, a stereotypic presentation of symptoms and course, and a relative gender specificity AN is possibly the most homogenous of all psychiatric disorders. The average point prevalence rate of AN is 0.3% in young females [1,2] and the lifetime prevalence is 2.2% among females [3]. The condition largely affects young adolescent females, with a female–male ratio between 10:1 and 4:1 [1,4].

### Medical complications

AN is often associated with medical complications resulting from starvation, purging and/or over exercising. Common signs and symptoms include cardiovascular complications such as bradycardia, prolonged QTc and orthostatic hypotension, loss of subcutaneous fat tissue, impaired menstrual function, hair loss, and hypothermia [5]. With improved nutritional status or with remittance of abnormal eating and purging behaviours, most pathophysiological complications are reversible. Nevertheless, some physical consequences of AN can be life-threatening, such as electrolyte imbalances, severe bradycardia, and hypotension. Moreover, nutritional deficiencies may increase the risk of cardiac arrhythmias and intercurrent infection. Some medical consequences of eating disorders may be irreversible or have later serious consequences on health, especially osteoporosis, growth retardation, malfunctioning of the reproductive system and neurobiological changes of the brain caused by malnutrition [6,7].

### Mortality, course and comorbidity

AN is associated with the highest rate of mortality among all mental disorders, with a crude mortality rate of 5,9% and a mortality rate of 5,6% per decade [8,9]. Other studies found mortality rates in the same range [10-15]. Steinhausen found a mean crude mortality rate of 5,0% [11]. Causes of death ranged from eating disorder complications to suicide. The majority of individuals with eating disorders reported suicidal thoughts and about 22% attempt to commit suicide [16,17].

In an extensive literature review Steinhausen showed that less than half (46,9%) of the surviving patients recover on average from AN, one-third (33,5%) improve partially, and in 20,8% (0-79%) the disease takes on a chronic course [11]. It had to be noted that the criteria used to define recovery and chronicity are very divergent. The studies reviewed, vary considerably in duration of follow-up (1–29 years), whereas outcome is influenced significantly by the duration of follow-up, with a higher mortality but also a tendency towards recovery with increasing duration of follow-up in surviving patients [11]. On the other hand, chronicity is associated with poor outcome, indicating that some cases of AN are indeed treatment refractory [18]. For the purpose of this article, we chose to define chronicity as an illness duration of five years or more.

Patients with AN have elevated rates of lifetime diagnoses of anxiety disorders, depressive disorders, obsessive-compulsive disorder (OCD), personality disorders and substance abuse disorders [6,7,18]. Comorbidity in eating disorders is substantial and contributes to a less favorable outcome of AN.

### Treatment options

The therapeutic options for AN consist of different treatment-approaches that focus on weight restoration, changes in behaviour and reducing the psychological features of AN. However, evidence-based treatment for AN is very limited. There is no category A evidence and only family interventions meet category B criteria according to the NICE-guidelines [19-22].

Psychotherapeutic interventions include cognitive behavioural therapy (CBT) and family therapy. A number of studies have reported that CBT after weight restoration could be effective in reducing the risk of relapse in adults with AN, but it is unknown what the efficacy is in underweight patients (see [23] for review). Variants of family therapy show a modest level of evidence with regard to efficacy in adolescents but not in adults.

SSRIs are ineffective in reducing AN symptoms or restoring weight and the American Psychiatric Association does not support the use of SSRIs in the management of underweight patients with AN [23] (Treatment of patients with eating disorders, APA 2006). There is some, however weak, evidence that the use of SSRIs may help in preventing relapse in weight restored patients [24,25]. Tricyclic antidepressants seem ineffective on weight gain or improvement of AN symptoms [26,27]. For atypical antipsychotics, there are only limited data available [28]. Bissada et al. conducted a double-blind, placebo-controlled trial in 34 patients with AN. Olanzapine treatment resulted in more rapid weight gain and improvement in obsessive symptoms [29]. Two other randomized, controlled trials with olanzapine showed similar results [30,31]. Another study, conducted in an inpatient setting, failed to show any benefit for olanzapine on weight and psychological symptoms [32]. These results indicate that olanzapine may be helpful in increasing weight and decreasing obsessive symptoms in chronic severe AN in outpatients [33], but practice guidelines do not recommend its routine use [34,35] (American Psychiatric Association 2006).

Unfortunately, there is no agreement on the definition of treatment-refractoriness in AN [36]. Refractory AN is a term used in clinical psychiatry to describe cases of AN not responding to typical modes of treatment, such as psychotherapy and psychopharmacology. Strober et al. (1997) and Herzog et al. (1999) found that the possibility of recovery in AN patients with an illness duration longer than 10 years is very low [37,38].

## Review

### Etiopathology of AN

The pathophysiology of AN remains unknown. It is unclear whether there is a primary disturbance of appetite, or whether the disturbed appetite is secondary to other phenomena, such as body image disturbance, or anxiety [39]. The aetiology of eating disorders is considered multifactorial, with involvement of genetic factors [40-45], neurobiological factors, and temperamental vulnerabilities such as negative emotionality, poor intraceptive awareness, perfectionism and obsessive-compulsive personality traits [41,46-48], that may interact with environmental factors resulting in an increased risk [39,49]. The neurobiology and neurocircuitries involved in AN are a main focus in today’s AN research. Of particular interest are the brain areas involved in registering the reward value and modulation of reward of food and the motivation to eat, considered to be located within the mesolimbic cortex and striatum [50,51]. Moreover, the brain areas involved in the cognitive control of eating and appetite, located in the dorsolateral prefrontal and parietal cortices may be involved in AN [39,49].

#### Neurotransmitters

Results from positron-emission tomography (PET) studies indicate differences between subjects recovered from AN and healthy subjects in serotonin and dopamine receptor activity, indicating dysregulation of these systems involved in mood, anxiety, appetite and impulse control [39,52].

Several studies showed alterations in the serotonergic (5-HT)-system in AN. For example, Kaye et al. (2009) reported elevated 5-HT metabolite levels, as well as elevated binding potential for postsynaptic 5-HT1a receptors and diminished binding potential for 5-HT2a receptors in recovered AN patients [53-55]. In contrast, ill AN patients were shown to have reduced amounts of the major 5-HT metabolite, 5-hydroxyindoleacetic acid (5-HIAA) [39]. Finally, it has been found that a dietary-induced reduction of tryptophan, the precursor of serotonin, is associated with decreased anxiety in AN patients [56]. Starvation may help AN patients to briefly reduce 5-HT activity and thus give symptom relief.

These alterations in 5-HT function may be related to AN symptoms regarding inhibition of appetite, generalized inhibition, anxiety and obsessions through stimulation of 5-HT1a receptors.

Several studies showed that ill and recovered AN patients also have altered striatal dopamine (DA) function. Kaye et al. (1999) found reduced levels of DA metabolites in the cerebrospinal fluid in ill and recovered AN patients [57]. Frank et al. (2005) found that patients recovered from AN had increased D2/D3 receptor binding potential in the ventral striatum (VS), indicating either increased D2/D3 receptor density or decreased extracellular DA, or both [58]. In addition, functional DA D2 receptor gene polymorphisms have been associated with AN [59] and subjects with AN have impaired visual discrimination learning, indicating altered DA neurotransmission in AN [60].

Disturbances in the DA-system may contribute to an altered response to reward and alterations in decision-making and executive control found in patients with AN.

Similar alterations of 5-HT and DA function are also found in other reward-related disorders like OCD, although the exact mechanisms and the interactions between these and other neurotransmitter systems are not clarified yet [61].

#### Neuroimaging

Imaging studies showed that in the acute phase of AN, patients have a reduced brain volume and enlargement of the cortical sulci and the ventricles [62-64]. Alterations in activity in the insula and the prefrontal, orbitofrontal, temporal, parietal and anterior cingulate cortices and the VS at rest and during symptom provocation were found [65-71]. These alterations may however be contributed to malnourishment and are mostly reversible with weight restoration.

However, functional imaging studies conducted in patients recovered from AN possibly reflect trait-related rather than state-related changes which could be an indication of the possible underlying neurobiological mechanisms in AN [72-74]. Several studies reported hypoperfusion of temporal, parietal, occipital and frontal regions in AN patients not correlated to their BMI [75-77]. It has also been reported that remitted as well as non-remitted AN patients have increased activations in brain regions implicated in the reward system (the medial prefrontal cortex and anterior cingulated cortex (ACC)) in response to food stimuli [78]. In a study of Wagner et al. (2007) no difference was found in ventral striatal response discriminating between positive and negative feedback in recovered AN patients, suggesting an impairment to identify the emotional significance of stimuli and the involvement of the brain reward system. Recovered AN patients also showed exaggerated activation of the caudate-dorsal striatum region and the dorsolateral prefrontal and parietal cortex, regions concerned with planning and consequences [79]. Zastrow et al. (2009) showed that impaired behavioral set shifting in AN is associated with hypoactivation in the ventral anterior cingulate-striato-thalamic loop and with hyperactivation of frontoparietal networks [80]. Another study by Wagner et al. (2008) showed a reduced BOLD signal response to sucrose in the anterior insula, the ACC and the striatum, indicating an altered incentive processing in the anterior insula [81]. All these studies implicate the involvement of the fronto-striatal circuitry in the neuropathogenesis of AN.

The insula is thought to be implicated in taste and its incentive value, interoceptive awareness and fear [49]. The ACC is associated with emotional processing, body image, self-monitoring, conflict resolution, and reward-based decision making [72,78,82]. The parietal lobe is functionally associated with disturbed body image and the low or absent insight in their condition, two of the core features of AN [67,72]. It is hypothesized that disturbances in interoceptive awareness, impairments of the ventral striatal pathways and an enhanced cognitive control (either inhibiting the reward system or compensating for primary deficits in limbic function) are involved in the pathogenesis of AN [39].

Because of the involvement of the reward-related neurocircuitries described above, the areas communicating between the limbic and the cortical systems, such as the nucleus accumbens (NAc) and the cingulate and insular cortices [39,83] may be of particular interest as possible target areas for future neurosurgical interventions.

### Deep brain stimulation

Deep brain stimulation (DBS) is an innovative and promising approach for the treatment of patients with treatment-refractory reward-related psychiatric disorders [84-87]. DBS is a reversible and adjustable neurosurgical treatment involving the implantation of electrodes that send electrical impulses to specific locations in the brain, selected according to the type of symptoms to be addressed and its putative neuroanatomical correlates [88,89].

Our center has experience with DBS in OCD, addiction, and major depressive disorder. In all these disorders, DBS targets reward related brain areas such as the NAc and the ventral capsule/ventral striatum (VC/VS). Target selection has evolved based on clinical results from earlier ablative procedures and DBS-studies, neuroimaging studies and theoretical considerations regarding the implicated neurocircuitries involved in these disorders [84,86,87,90]. The current working hypothesis is that DBS inhibits or functionally overrides pathological network hyperactivity in several treatment-resistant psychiatric disorders. Our research group showed efficacy for DBS in OCD targeted at the NAc normalizing NAc activity, reducing excessive connectivity between the NAc and prefrontal cortex, and decreasing frontal low-frequency oscillations during symptom provocation in patients with OCD. These findings taken together suggest that DBS is able to reduce maladaptive activity and connectivity of the stimulated region and to restore disease-related brain networks to a healthy state [91].

To consider a psychiatric disorder as a possible new indication for DBS and a particular patient as candidate Denys (2008) suggested the following criteria [92]:

Disorder-related criteria:

(a) A general agreement on the neuropsychiatric nature of the disorder.

(b) A proven relationship with a dysfunctional brain circuitry.

(c) Objectively measurable symptoms.

Patient-related criteria:

(a) Presence of very severe symptoms and considerable suffering.

(b) Absence of available effective treatments.

(c) Potential to regain reasonable functioning and integration in society.

### DBS in AN

Considering 1) the clear neurobiological correlates of the disorder, 2) the homogeneity of the disorder, 3) the severity of AN, its complications and high mortality rates, 4) the fact that AN takes on a chronic course in a considerable percentage of patients and the fact that, up to date, evidence based treatment for AN is very limited, it is crucial to investigate new treatment options for AN that focus on influencing the underlying neurobiological mechanisms of the disease rather than focus on weight restoration alone. In this review article we want to propose DBS as a possible new treatment option for patients with chronic, treatment-resistant AN.

For a long time, neurosurgery targeted at various brain areas (mostly leucotomy) has been considered a last resort treatment for AN. While reviewing the literature on neurosurgical procedures in AN, we found most articles reported weight gain and sometimes other symptomatic improvements. However, there was much heterogeneity as well as missing data on patient selection, follow-up and outcome measurements. Therefore, clinical outcome appears at least to us, somewhat inconclusive [93-101]. In a case-report of Barbier et al. (2011) a successful anterior capsulotomy in comorbid AN and OCD is described, resulting in normalization of eating pattern and weight and a significant decrease of food-related obsessive compulsive symptoms after three months [102].

To date, there are very limited data on the effect of DBS in the treatment of AN. There are two case reports, a case series of four patients and a pilot study published on DBS in AN (see Table 1). Israël et al. (2010) described a patient treated with DBS in the subgenual cingulate cortex, part of the ACC, for severe refractory depression, whose co-morbid eating disorder showed lasting remission, consisting of a normalisation of BMI (19,1 kg/m^2^) and Eating Attitudes Test-26 at 2-year follow-up. It must be noted that pre-surgery this patient also had periods of (partial) recovery from her eating disorder, and that her BMI pre-surgery was 20,9 kg/m^2^[103]. A more recent case report by McLaughlin (2012) described improvements in AN symptoms following DBS of the VC/VS for intractable OCD [104]. The first study (case series) on DBS in AN was conducted by Sun et al. from the Shanghai group. Preliminary results reported an average of 65% increase in body weight at 38-month follow-up in four adolescent patients with AN treated with DBS of the NAc, showing that DBS might be a valuable option for weight-restoration in AN [105]. Very recently, Lipsman et al. (2013) published the results of a phase-1 pilot trial of subcallosal cingulate (ACC) DBS in six adult patients with treatment-refractory AN. They found that DBS was relatively safe in this population and found to result in improvements of mood, anxiety, affective regulation and anorexia-related obsessions and compulsions in four patients. Furthermore, at a 9 month follow up period improved BMI’s compared to the estimated historical baseline BMI’s in three patients were found [101,106].

**Table 1:** DBS in AN

**Study**	**n**	**DBS target**	**Result**
Israel (2010)	1	Subgenual cingulate cortex	DBS for treatment resistant major depression. Co morbid eating disorder-NOS in lasting remission (normalisation of scores on the Eating Attitudes Test-26 and Eating Disorders Examination; normalisation of weight (BMI 19,1 kg/m^2^) at 2 and 3 year follow-up)
McLaughlin (2012)	1	Ventral capsule/ventral striatum	DBS for treatment resistant OCD. Improvements in AN symptoms consisting of less distress about caloric intake and weight (assessment tools and length of follow-up not mentioned; BMI pre-surgery 18,5 kg/m^2^, post-surgery 19,6 kg/m^2^)
Sun et al. *in* Wu et al. (2012)	4	Nucleus accumbens	Average of 65% increase in body weight at 38-month follow-up (average baseline BMI: 11,9 kg.m^2^; average BMI at follow-up: 19,6 kg/^2^); restoration of the menstrual cycle (n = 4); regaining school functioning (n = 3); remission of AN according to the DSM-IV (n = 4)
Lipsman et al. (2013)	6	Subcallosal cingulate	Relatively safe (1 serious adverse event), improvement of BMI compared to historical baseline (n = 3) at 9 month follow-up (average baseline BMI: 13,7 kg/m^2^; average BMI pre-surgery: 16,1 kg/m^2^; average BMI at 9 month follow-up: 16,6 kg/m^2^). Improvements in mood, anxiety, affective regulation and anorexia-related obsessions and compulsions (the latter assessed with the Yale-Brown-Cornell eating disorder scale) at 6 month follow-up; Improvements in quality of life (n = 3)

#### Possible DBS targets for AN

As stated before, many parallels can be drawn with regard to symptoms of AN and OCD [107]. Moreover, there is a considerable overlap between the neurocircuits implicated in OCD and eating disorders, suggesting a possible etiological relationship between the two disorders. Both disorders consist of repetitive thoughts and preoccupations about a feared stimulus, followed by a negative emotion, than followed by compensatory behaviours. Like OCD, AN can be considered a compulsivity disorder. Compulsivity encompasses the repetitive, irresistible urge to perform a behavior, the experience of loss of voluntary control over this intense urge, the diminished ability to delay or inhibit thoughts or behaviors, and the tendency to perform repetitive acts in a habitual or stereotyped manner [108]. Research showed that AN is associated with impairments in set shifting and behavioural response shifting [80,109]. Furthermore, there is a high rate of comorbidity between eating disorders and anxiety disorders [110-112]. Kaye et al. reported that 41% of AN patients have a lifetime diagnosis of OCD [111]. Alterations in the activity of cortico-thalamo-striatal circuits similar to those found in OCD and other compulsivity disorders have commonly been found in AN, as described above [39,72,113-115].

Given the similarities in symptomatology and associated neurocircuits between OCD and AN, the established efficacy of DBS in OCD [87], and the neurobiological correlates of AN as described above, we hypothesize that DBS of the NAc and other areas associated with reward, e.g. the ACC, might be effective in patients with chronic, treatment refractory AN, providing not only weight restoration, but also significant and sustained improvement in AN core symptoms and associated comorbidities and complications. Possible targets for DBS in AN are the ACC, the ventral anterior limb of the capsula interna (vALIC) and the VS (consisting of the ventral caudate nucleus and the NAc).

#### Suggestions for in- and exclusion criteria

As DBS in AN is an experimental treatment whose efficacy still needs to be established, it would be logical to initially include only patients with a chronic course who are treatment-resistant and have a poor prognosis. As stated before, there has thus far been no agreement on the definitions of recovery and treatment-refractoriness in AN [35]. We suggest to define treatment-refractoriness as a lack of response to two or more typical modes of treatment, including one hospital admission or inpatient treatment in a clinic specialized in the treatment of eating disorders, and an illness duration of five years or more. We suggest inclusion and exclusion criteria for studies investigating the efficacy of DBS in AN similar to those suggested by Wu et al. (2012) and Lipsman et al. (2013) [105,106]:

Inclusion criteria

•Primary diagnosis: Anorexia Nervosa (restricting or purging type) according to the DSM-IV criteria based on a psychiatric interview.

•Chronicity, defined by an illness duration > 5 years.

•Disabling severity with substantial functional impairment according to the DSM-IV criterion C and a Global Assessment of Function (GAF) score of 45 or less for at least two years.

•Treatment refractoriness, defined as lack of response to two or more typical modes of treatment, including one hospital admission or inpatient treatment in a specialized clinic.

•Weight <85% of ideal body weight (and/or BMI < 17,5).

•Age: 21–65 years old.

•Able to fully understand the consequences of the procedure capable to make his or her own choice without coercion and able to give written informed consent.

Exclusion criteria

•Unstable physical condition (severe electrolyte disturbances, cardiac failure, other physical contraindications for surgery/anesthesia).

•A treatable underlying cause of anorexia/underweight.

•An active neurological disease like Parkinson’s disease, dementia, tic disorder or epilepsy.

•Schizophrenia/history of psychosis, bipolar disorder.

•Alcohol or substance abuse (including benzodiazepines) during the last 6 months.

•Antisocial personality disorder.

•Standard MRI scan exclusion criteria (pregnancy, pacemaker and metals contraindicated for MRI except for the DBS implantation and stimulator itself).

#### Safety considerations

In general, potential risks involved in DBS include the risks associated with the surgical procedures, including the small risk (< 1%) of intracranial haemorrhage or infection and the associated neurological consequences [116]. In addition, some patients may show some temporary neurological symptoms (e.g. eye movement abnormalities) that generally disappear spontaneously or after some fine-tuning of the stimulator.

Patients with AN are predisposed to significant risk of multi-organ dysfunction related to starvation and purging. This can have implications on mortality and morbidity associated with anesthetic complications during DBS implantation [117]. Therefore, a thorough pre-operative anaesthetic assessment and evaluation is required to assist the planning of safe peri-operative care. Patients should be rehydrated adequately and any deranged electrolyte levels should be corrected pre-operatively. There is an increased risk of intra-operative hypothermia. Therefore measures should be taken to keep the patient warm during surgery. Doses of most (anaesthetic) drugs should be adjusted for weight. Patients are particularly susceptible to nerve palsies due to their cachexia and loss of cushioning subcutaneous tissue. Therefore they must be placed carefully on the operating table. During the operation, ECG-changes and potassium levels should be monitored carefully to minimize the risk of arrhytmias. To minimize the overall increased risks associated with anaesthesia the weight and somatic condition will be maximally optimized prior to surgery.

Lipsman et al. (2013) reported several adverse events in their pilot study on DBS in AN, with one serious DBS-related adverse event (seizure during programming) and the other serious adverse events being related to the underlying illness. One patient in this study developed hypophospataemia and a refeeding delirium [106]. It is expected that weight increase following treatment with DBS will be gradual rather than sudden and excessive. Other DBS studies, for example in OCD and depression, showed that improvement of symptoms takes several months [85-87,106]. However, in case of rapid weight gain following successful treatment with DBS, there is a risk of development of a refeeding syndrome [118]. Therefore, AN patients treated with DBS should be advised to increase their food intake gradually and under supervision of a dietary consultant. The somatic condition and the potential development of a refeeding syndrome should be closely monitored by a physician.

## Conclusions

AN is a serious psychiatric disorder with high rates of morbidity, comorbidity and mortality, that takes on a chronic course in a considerable percentage of patients. Since evidence-based treatments are scarce, it is crucial to investigate treatment options based on underlying neurobiological mechanisms of the disease.

The fronto-striatal circuitry, in particular the insula, the VS and the prefrontal, orbitofrontal, temporal, parietal and anterior cingulate cortices, appear to be implicated in the etiopathogenesis of AN. Thus, the areas communicating between the limbic and the cortical systems, such as the NAc and the cingulate and insular cortices may be of interest as target areas for future neurosurgical interventions.

DBS had the advantage over ablative neurosurgery in being reversible and adjustable and studies show that DBS is able to reduce maladaptive activity and connectivity of the stimulated region and to restore disease-related brain networks to a healthy state. Given the overlap in symptomatology and associated neurocircuits between reward-related disorders like OCD and AN, and the established efficacy of accumbal DBS in OCD, we hypothesize that DBS of the NAc and other areas associated with reward, e.g. the ACC, might be an effective treatment for patients with chronic, treatment refractory AN, providing not only weight restoration, but also significant and sustained improvement in AN core symptoms and associated comorbidities and complications. Possible targets for DBS in AN are the ACC, the vALIC and the VS (consisting of the ventral caudate nucleus and the NAc).

Larger studies with primary outcome aimed at sustained core symptom reduction and weight restoration are necessary. Preferably, studies should be conducted with a double blind cross-over design with active and sham stimulation. Furthermore, functional effects of DBS in AN should be explored by evaluating neuropsychological parameters and by using neuroimaging techniques.

In our opinion, the seriousness of the disorder and the clear neurobiological substrates of AN justify considering an invasive procedure like DBS as a treatment-option for chronic, treatment-refractory AN. When carefully selecting the stimulation target, using clear in- and exclusion criteria and closely monitoring the safety aspects of DBS in this population, and in the meanwhile thoroughly investigating the clinical and functional effects, DBS could be promising in attacking the core symptoms of AN and contribute to the knowledge of the intriguing pathophysiological mechanisms of AN.

## Competing interests

The authors declare that they have no competing interests.

## Authors’ contributions

MO drafted the manuscript. JS revised the manuscript critically for important intellectual content. EN did a literature search for neurosurgery on AN. DD made substantial contributions to the structure and design of the manuscript and revised it critically. All authors read and approved the final manuscript.

## Pre-publication history

The pre-publication history for this paper can be accessed here:

http://www.biomedcentral.com/1471-244X/13/277/prepub
